# Does targeted temperature management at 33 °C improve outcome after cardiac arrest?

**DOI:** 10.1097/MCC.0000000000001214

**Published:** 2024-10-21

**Authors:** Markus B. Skrifvars, Benjamin S. Abella

**Affiliations:** a Department of Emergency Care and Services, University of Helsinki, Helsinki University Hospital, Finland; b Mount Sinai Professor and System Chair, Department of Emergency Medicine, Icahn School of Medicine at Mount Sinai

**Keywords:** cardiac arrest, ischemia–reperfusion injury, postarrest care

## Abstract

**Purpose of review:**

Following successful resuscitation from cardiac arrest, a complex set of pathophysiologic processes are acutely triggered, leading to substantial morbidity and mortality. Postarrest management remains a major challenge to critical care providers, with few proven therapeutic strategies to improve outcomes. One therapy that has received substantial focus is the intentional lowering of core body temperature for a discrete period of time following resuscitation. In this review, we will discuss the key trials and other evidence surrounding TTM and present opposing arguments, one ‘against’ the use of postarrest TTM and another ‘for’ the use of this therapeutic approach.

**Recent findings:**

Targeted temperature management, has been a topic of enormous controversy, as recently a number of clinical trials show conflicting results on the effect of TTM. Fundamental questions, about the dosing of TTM (e.g. use at 33 °C versus higher temperatures), or the use of TTM at all (as opposed to passive fever avoidance), remain active topics of global discussion. Systematic reviews on this topic also show variable results.

**Summary:**

There are several arguments for and against the use of TTM targeting 33 °C for alleviating brain injury after cardiac arrest. More studies are on the way that will hopefully provide more robust evidence and hopefully allow for consensus on this important topic.

## INTRODUCTION

The use of therapeutic hypothermia, more recently known by the broader term targeted temperature management (TTM), has been long considered as a potential strategy to improve outcomes from cardiac arrest. The proposed mechanisms of action from lowering core body temperature include the mitigation of cellular injury by stabilization of mitochondrial function and attenuation of apoptotic pathways, reduction of inflammatory cytokine levels, and lessening of cerebral oedema. Supported initially by laboratory studies that employed animal models of cardiac arrest, clinical methods to apply TTM were then developed and eventually tested in two randomized clinical trials of patients who experienced out-of-hospital cardiac arrest (OHCA). These trials both demonstrated a survival benefit from TTM, leading international guidelines to recommend the therapy as an option for patients following arrest who exhibit signs of neurologic injury [e.g. lack of ability to follow commands after return of spontaneous circulation (ROSC)].

In more recent clinical investigations, the outcome benefit of TTM has been called into question, leading to widespread controversy about whether TTM should be employed at either 33 or 37 °C in the initial care following arrest resuscitation, or even used at all. This review will frame the evidence surrounding postarrest TTM as a ‘pro/con’ discussion, with the first section, prepared by one author (B.S.A.) making the argument in favour of TTM use for patients following cardiac arrest resuscitation, and the second section making the argument, that TTM is not supported by the body of evidence at the present time, prepared by another author (M.B.S.). 

**Box 1:**
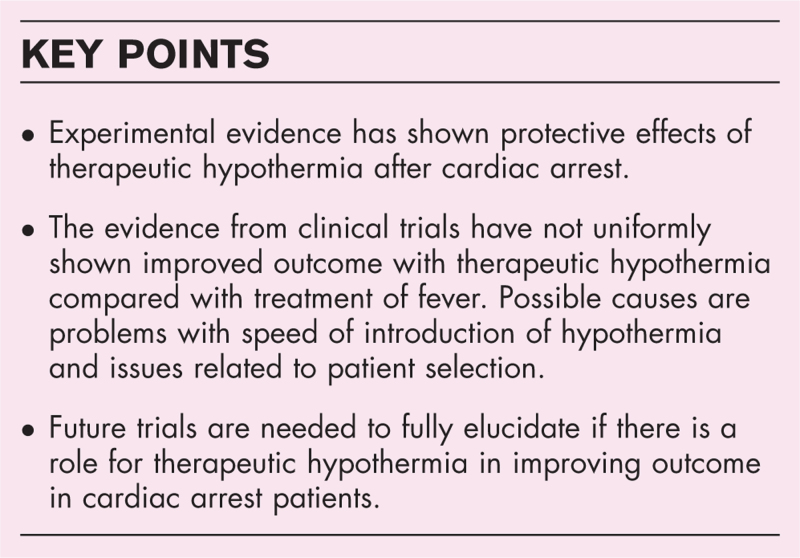
no caption available

## PROS: PRESENTED BY BENJAMIN S. ABELLA

The use of postarrest TTM is well grounded in extensive laboratory evidence [1–4], demonstrating a survival benefit of TTM and perhaps more strikingly, a strong dose–effect relationship [3,4], in which lower target temperatures and longer durations of TTM are associated with improved outcomes. In one such example, a rat model of arrest was employed to demonstrate both improved outcome and evidence of lessened brain injury with 48 h of postarrest TTM compared with 24 h [3]. In other example, a porcine model was used to demonstrate that TTM to lower temperatures led to lessened injury compared with TTM at higher temperatures [4]. The laboratory investigative literature on TTM is vast, and only a small selection of studies are represented here; the great majority show a benefit of TTM with strong mechanistic underpinnings.

Following the two landmark clinical trials of 2002 [5,6], two randomized trials of postarrest TTM were conducted that raised questions about the benefit of TTM [7,8]. These were led by an investigative group in Sweden, and included a largely European patient population. These studies, known as the TTM1 and TTM2 trials, yielded null results under each of their trial frameworks. In the TTM1 trial [8], postarrest survival was equivalent when patients were treated with TTM to 33 or 36 °C; in the TTM2, postarrest survival was indistinguishable when patients were treated with TTM to 33 °C or a pragmatic normothermia control strategy of lower than 37.8 °C [7]. However, it is important to note that the TTM1 and TTM2 investigations involved a narrow clinical population of postarrest patients that may not be easily generalizable to the larger range of postarrest patients. In both studies, the percentage of bystander CPR was over 70%, the vast majority of patients in both studies experienced witnessed cardiac arrest, and enrolled patients exhibited low rates of shock and/or hemodynamic instability. Furthermore, a remarkably large proportion of patients (over 30% in both studies) received percutaneous coronary intervention for presumed acute coronary syndrome aetiologies of arrest; whether these studies might apply to patients with more complex and injurious pathophysiologies of arrest remains an open question. Very few patients were enrolled in the United States or even outside of northern Europe in these studies, raising additional questions regarding generalizability based on local emergency medical service and hospital practices in these locations compared with very different clinical environments in the United States and elsewhere [9].

Another multicentre randomized trial of postarrest TTM was conducted in France that indirectly addressed this question about whether the TTM1 and TTM2 trials enrolled patients who were not sufficiently injured to require aggressive TTM therapy. In this French study, known as the HYPERION trial [10], patients were randomized to 33 vs. 37 °C following nonshockable rhythm arrest (asystole and pulseless electrical activity). Overall survival was substantially lower in this work, and patients exhibited a much higher degree of postarrest injury. In this population, TTM to 33 °C provided statistically significant neurologic outcome benefit compared with 37 °C. It is therefore reasonable to hypothesize that the benefit of TTM is directly associated with the degree of injury in any given patient, and perhaps to abandon a potentially life-saving therapy for all patients without this consideration may deny many injured patients an opportunity for improved outcomes. Furthermore, it is important to note that the TTM1 and TTM2 trials were neutral, without any substantial injury or increased mortality from the use of TTM. When a critical care provider is confronted with a postarrest patient with a high risk of mortality or crippling brain injury, and is faced with the balance of evidence (three randomized trials showing survival benefit, no clinical trials showing clinical harm, and two neutral studies), it seems odd to deny a potential therapy with strong mechanistic evidence and rationale based on two studies with neutral results. In the world of oncology, for example, therapeutic agents are used routinely with a similar mixed evidence base; provided there is no evidence of substantial harm, clinicians recommend chemotherapeutic regimens under the assumption that a small number of patients may derive important benefits.

Subsequent observational studies have supported this concept that TTM dose may very much hinge on the specific injury profiles of the treated patients. In work from Callaway *et al.*[11], postarrest patients were categorized by global injury profiles into four categories using the Pittsburgh Cardiac Arrest Category (PCAC) paradigm. Patients recognized as exhibiting lesser postarrest injury had better outcomes when treated with TTM at 36 °C, but patients with more significant injury had better outcomes when treated with TTM at 33 °C. In other observational work from Japan [12], a similar result was derived when examining patients treated at 33 or 36 °C. Moderately injured patients, as defined using a composite injury score known as rCAST, had significantly better outcomes when managed at 33 °C compared with 36 °C. In a third study from the Netherlands, patients with higher degrees of brain injury as characterized by electroencephalography had better outcomes when treated with TTM to 33 °C [13]. Taken together, these three studies, using very different methodologies in different clinical environments, all provide similar robust results: the use of TTM in more injured patients may provide substantial benefit, whereas the use of TTM in less injured patients may not.

Given a therapy that has a highly favourable side effect profile with no evidence of lessened survival when employed, and strong evidence that in some patients, significant benefit can be derived, the argument against TTM use appears largely process-oriented and academic; why provide a therapy, with associated work and expense, when several large studies have a null result? This argument neglects the strong mechanistic evidence of benefit, the balance of clinical trials that find outcome improvement with TTM, and the observational data that suggest that the path forward may involve aggressive TTM for those who exhibit serious injury. Future studies of TTM are required; specifically, trials that involve judicious tailoring of TTM therapy based on physiologic markers of injury. Without such studies, the controversies surrounding TTM will continue based on trials that involve overly broad enrolment criteria that risk oversimplification of a complex disease process and the inability to statistically identify patients who might benefit from this important therapy [14].

## CONS: PRESENTED BY MARKUS B. SKRIFVARS

As described in previous sections, two randomized trials established the clinical use of postarrest TTM in 2002 [5,6]. Overall, only around 350 patients were included in these two trials, which showed improved outcomes with therapeutic hypothermia compared with no therapeutic hypothermia. The number of patients included in these trials may seem small by today's standards, but at the time, this was a fairly large number compared with many other critical care interventions, such as early goal-directed therapy, intensive glucose control and the use of decompressive craniectomy in treatment-resistant intracranial hypertension in the ICU [15–17]. These two studies received much attention, and quite quickly, therapeutic hypothermia for a duration of 12–24 h became standard clinical practice and was recommended by international guidelines [18]. This was of course also supported by experimental evidence supporting hypothermia as a means to alleviate brain injury after cardiac arrest [19,20]. In retrospect, these two studies can be criticized. The Hypothermia after Cardiac Arrest (HACA) trial was stopped prematurely, increasing the risk of chance findings. In addition, the duration of the ICU stay was not reported, and few data are available on how the prognosis was determined in the included patients. At the time of the HACA trial, there was little guidance on prognostication, and as withdrawal of life-sustaining therapies is a major contributor to outcome, omission of these data from the manuscript is a limitation [6]. With regard to the performance of therapeutic hypothermia, it is noteworthy that the median time to target temperature from ROSC was 8 h (interquartile range 4–16 h), and in 15% of the patients, the target temperature was not achieved. Nonetheless, there was a significant difference in both mortality and functional outcome. The number needed to treat (NNT) for preventing death was 7. However, importantly, the 95% confidence interval for the NNT was 4–33, which clearly suggests the uncertainty of the effect size of the intervention. In addition, in the HACA trial, only 8% of the screened patients were included. The study by Bernard *et al.* was small, and randomization was based on the day of the week.^1^ These uncertainties prompted the TTM, TTM2 and Therapeutic Hypothermia after Cardiac Arrest in Nonshockable Rhythm (HYPERION) trials [7,8,10].

In the TTM trial, which included more than 900 patients, no difference in outcome was found in patients treated with a target temperature of either 33 or 36 °C [8]. In the TTM2 trial with 1800 patients, no difference was found in either functional outcome or mortality between therapeutic hypothermia and nontherapeutic hypothermia patients [7]. If anything, there was a 2% difference in nonsignificant higher mortality in the therapeutic hypothermia group compared with the nontherapeutic hypothermia group. In addition, there were more cardiac side effects in the therapeutic hypothermia group than in the nontherapeutic hypothermia group. The HYPERION trial focused on patients with a nonshockable initial rhythm, the majority being OHCA patients, but some in-hospital cardiac arrest (IHCA) patients were included [10]. No difference in mortality was found between the therapeutic hypothermia and nontherapeutic hypothermia groups, but the therapeutic hypothermia groups had more patients with favourable functional outcomes. However, almost all this difference was seen in the IHCA patient group (10% absolute difference). The difference was much smaller in the OHCA cases (2% difference). The effect of therapeutic hypothermia on outcome after IHCA was also studied in a German trial that included 250 patients, which showed no difference in either mortality or functional outcome [21].

Typical criticisms of all the recent large multicentre trials have varied from the inclusion of patients with less severe brain injury, slow induction of hypothermia, delays in reaching the target temperature and the experience of the study centres [22^▪^]. However, the critiques appear ill-founded. The patients had mean ROSC delays of around 25 min, and mortality was around 50%. The time to reach the target temperature was around 5–6 h, which is clearly within regular clinical practice outside clinical trials [23^▪▪^]. When the author lists of these studies are examined, it appears quite clear that the centres taking part represent high-performing centres that have conducted research in both postcardiac arrest management and intensive care in general [7,8]. Further, an important part of all the recent trials has been the use of a clear prognostication algorithm for identifying patients with a poor outcome and the use of physicians blinded to the intervention to perform the outcome assessments.

Systematic reviews on whether therapeutic hypothermia improves outcome show divergent results [23^▪▪^,^24^^▪▪^,^25^]. Larger treatment effects were found in older single-centre studies. Some included studies were only published as abstracts, making a thorough bias assessment difficult [24^▪▪^,^26^]. The number of patients included in the later trials outnumbered the earlier trials. Interestingly, the more recent trials also appear to be of higher quality with regard to the therapeutic hypothermia intervention: target temperature was reached faster, and more invasive and noninvasive feedback-controlled therapeutic hypothermia devices were used [27–29]. The only subgroups in which there appeared to be a suggestion of improved outcome based on an individual patient data meta-analysis appeared to be those without bystander-initiated life support [30]. In addition, several observational cohort studies have reported outcomes before and after local changes in TTM practices. Some studies have shown a difference in outcome, whereas others have not [31,32^▪^].

Nonetheless, further trials on this topic are needed. Intra-arrest hypothermia combined with prolonged therapeutic hypothermia can well be studied further, and new trials are underway [33–35]. However, if these interventions are to be widely adopted and recommended in international guidelines, their clinical effects must be shown in pragmatic large multicentre trials. It appears clear that we have been too optimistic with regard to the effect sizes of the studies’ interventions [25]. We owe it to our patients to base our clinical treatment decisions on results from clinical trials evaluating the intervention in realistic real-life scenarios, replicating the settings in which they are to be used. Relying on experimental animal evidence is simply not good enough, and the same applies to other drugs and procedures in medicine. Therefore, therapeutic hypothermia should only be used currently as part of a clinical trial. Currently, no high-quality multicentre data to support the use of therapeutic hypothermia as part of clinical practice exist, and therefore it should have no or at least a very minimal role in current postcardiac arrest care guidelines [36]. Further research efforts in this area should continue, and if the evidence base evolves, then the guidelines should change accordingly.

## CONCLUSION

**FIGURE 1:**
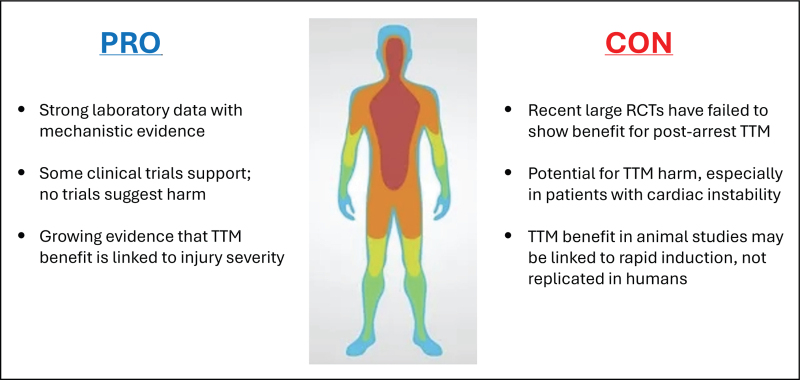
Illustration of select arguments in favour and against the routine use of targeted temperature management for postarrest care.

The study of TTM as a therapy during postarrest care has proven to be an enormous challenge, due to issues of timing, logistics, illness severity and patient heterogeneity. Although laboratory science has repeatedly shown a clear survival benefit following cardiac arrest when TTM is used (and indeed, with a strong dose–effect relationship), similar results have been elusive in clinical trials, with some trials showing benefit and others showing no clinical effect when TTM is employed (Fig. 1). Although we have presented arguments both against and for the use of TTM following arrest, it is clear that further work is required, particularly in the domain of patient selection and matching therapeutic strategies with illness severity. We hope that additional trials will provide key data to determine which patients, if any, might benefit from TTM and have a greater chance to return to meaningful life after cardiac arrest.

## Acknowledgements

*None.*

### Financial support and sponsorship

*None.*

### Conflicts of interest

*M.B.S. reports speaker's fees from BARD Medical (Ireland) in 2021 and 2022. B.S.A. reports research funding from NIH, DOD and Becton Dickinson. B.S.A. reports consulting payments from Neuroptics, Beckton Dickinson and Zoll.*

## REFERENCES AND RECOMMENDED READING

Papers of particular interest, published within the annual period of review, have been highlighted as:

▪ of special interest
▪▪ of outstanding interest
